# The impact of armed conflict on vaccination coverage: a systematic review of empirical evidence from 1985 to 2025

**DOI:** 10.1186/s13031-025-00708-7

**Published:** 2025-10-14

**Authors:** Tyler Y. Headley, Christopher Wiley Shay, Yesim Tozan

**Affiliations:** 1 New York University Abu Dhabi, Abu Dhabi, United Arab Emirates; 2 Ash Center for Democratic Governance and Innovation, Harvard Kennedy School, Harvard University, Boston, MA USA; 3 T.H. Chan School of Public Health, Harvard University, Boston, MA USA; 4 School of Global Public Health, New York University, New York, NY 10003 USA

## Abstract

**Background:**

Armed conflict disrupts health systems and undermines routine immunization, contributing to excess morbidity and mortality. This systematic review examines empirical evidence on the impact of armed conflict on vaccination services and coverage, identifying patterns of disruption across geographic settings and conflict types.

**Methods:**

This study followed PRISMA guidelines and was registered in PROSPERO (CRD420251064804). We searched seven databases for peer-reviewed and grey literature (1985–2025) reporting quantitative comparisons of vaccination coverage before and after conflict onset, or between conflict-affected and unaffected populations. Screening and data extraction followed standardized systematic review protocols, with dual validation of a subset of studies. Due to methodological heterogeneity across studies, a meta-analysis was not conducted.

**Results:**

Of 8,043 citations screened, 33 met the inclusion criteria. Most focused on child immunization in settings across the Eastern Mediterranean (15, 45%) and African (12, 36%) regions. Data sources included household surveys (22, 67%) and health system records (8, 24%). Conflict exposure was most commonly measured using battle-related deaths (15, 45%). Analyses employing individual-level data were most common (10, 30%), followed by subnational administrative data (9, 27%). Nearly all studies (31, 94%) were observational or quasi-experimental. In 28 (85%) studies, conflict was associated with reduced vaccination coverage, sometimes exceeding 20% points for vaccines such as BCG, DTP, and polio. Declines were most pronounced in settings with civil war and moderate to high conflict intensity. Two studies reported localized increases in vaccination coverage, possibly due to targeted humanitarian interventions. Effect estimates were larger in studies using national or administrative-level data compared to those using household-level data, underscoring methodological variation as a key contributor to heterogeneity in reported impacts.

**Conclusions:**

Armed conflict is consistently associated with substantial declines in childhood vaccination coverage, most pronounced in civil war and military occupation settings and across conflicts with moderate-to-high annual BRDs. Regional disruptions were especially severe in the Eastern Mediterranean and sub-Saharan African regions. We found substantial variation in estimated effect sizes across analytic units (individual, household, region, country), suggesting that more aggregated data may better capture the broader impact of conflict on vaccination rates. Future research should incorporate standardized conflict and vaccination metrics to improve the generalizability of findings.

**Supplementary Information:**

The online version contains supplementary material available at 10.1186/s13031-025-00708-7.

## Introduction

Armed conflict—defined as violence between at least two parties, with at least one being formally organized and resulting in a minimum of 25 battle-related deaths (BRDs) in a calendar year [1]—remains a persistent driver of global health disruption [2]. In 2022 alone, more than 237,000 people died from organized violence, marking the deadliest year for conflict since the 1994 Rwandan genocide, amid a resurgence of both state-based and internationalized intrastate warfare [3]. By undermining access to basic preventive and curative services, these conflicts exacerbate existing health inequalities and disproportionately affect vulnerable populations, particularly young children and women [4, 5].

Among the most critical yet overlooked impacts of conflict is its disruption of routine immunization [6]. Vaccination services are uniquely vulnerable to breakdown during armed conflict due to population displacement, destruction of infrastructure, health worker shortages, and both the disruption and collapse of surveillance and supply chains [7]. While post-conflict vaccination campaigns—particularly for measles and polio—are occasionally deployed to great effect [8, 9], these catch-up campaigns are often delayed or limited in scale or coverage, leaving cohorts of children unprotected during and after periods of conflict [9–11]. These disruptions undermine global immunization efforts and targets—including the United Nations’ Sustainable Development Goal 3.b, WHO’s Immunization Agenda 2030, and the Global Polio Eradication Initiative—particularly as vaccine-preventable diseases resurge and coverage regresses in conflict-affected and politically unstable areas [12].

Despite the central role of immunization in controlling vaccine-preventable diseases, empirical literature assessing the impact of conflict on vaccination coverage remains surprisingly limited. Only 15% of studies evaluating health service delivery in conflict zones report coverage data, with even fewer reporting vaccination coverage estimates. Most of these studies focus narrowly on measles and polio immunization, showing considerable variation in findings [13]. While some isolated improvements in healthcare-seeking behavior have been observed under specific conditions, the prevailing evidence points to declines in service delivery and use during and after conflict [14]. This systematic review synthesizes global evidence on the effects of armed conflict on vaccination coverage, identifying patterns of disruption across geographic settings and conflict types.

## Methods

This systematic review is registered with PROPSERO (registration number CRD420251064804) and follows PRISMA standards. Its objective is to identify the empirical impact of violent conflict on vaccination coverage within affected populations.

### Search strategy

A systematic review of published literature from 1 st January 1985 to 7th February 2025 was conducted using Pubmed, Medline, Global Health, Scopus, Web of Science Core Collection, and the WHO Global Index Medicus. Predefined keywords were also searched in Google Scholar to capture relevant gray literature. The full search strategy is provided in the additional file (Additional Table 2).

### Eligibility criteria

To be included, studies needed to meet the following criteria based on the PICO framework (Population, Intervention, Comparator, and Outcome): [1] the population experienced armed conflict; [2] the study compared vaccination coverage rates before and after conflict onset or between conflict-affected and unaffected populations; and [3] empirical data were presented on the effects of conflict on vaccination coverage rates. We excluded systematic and other literature reviews, editorials, commentaries, first-person narratives, newspaper and magazine articles, guidelines, and qualitative studies exploring reasons why people in conflict zones did not get vaccinations. Studies with a primary focus on internally displaced persons or individuals residing in refugee camps were also excluded to better attribute immunization trends to conflict exposure in the general population rather than to displacement-related factors.

### Screening and data extraction

Search results were exported from each database and de-duplicated using the Rayyan systematic review software [15]. Screening was conducted in two stages: titles and abstracts were initially reviewed by a single reviewer, followed by data extraction using a standardized Excel template. Extracted data included information on study characteristics, conflict exposure, vaccination data, quantitative findings, analytical methods, and reviewer comments. A second reviewer validated the data extracted on a subset of studies, and any discrepancies were resolved by consensus. Studies published in languages other than English were translated using Google Translate prior to being screened for eligibility. A meta-analysis was not conducted due to substantive heterogeneity in study settings and methods. The methodological quality of included studies was evaluated independently by one reviewer using the appropriate Joanna Briggs Institute (JBI) standardized critical appraisal tools [16], and was validated by a second reviewer. One question from the JBI tool for quasi-experimental studies was excluded as it was not applicable as no eligible studies included longitudinal patient data.

### Patient and public involvement

Patients and the public were not involved in the design, conduct, reporting, or dissemination plans of this systematic review.

## Results

### Characteristics of the included literature

**Fig. 1:**
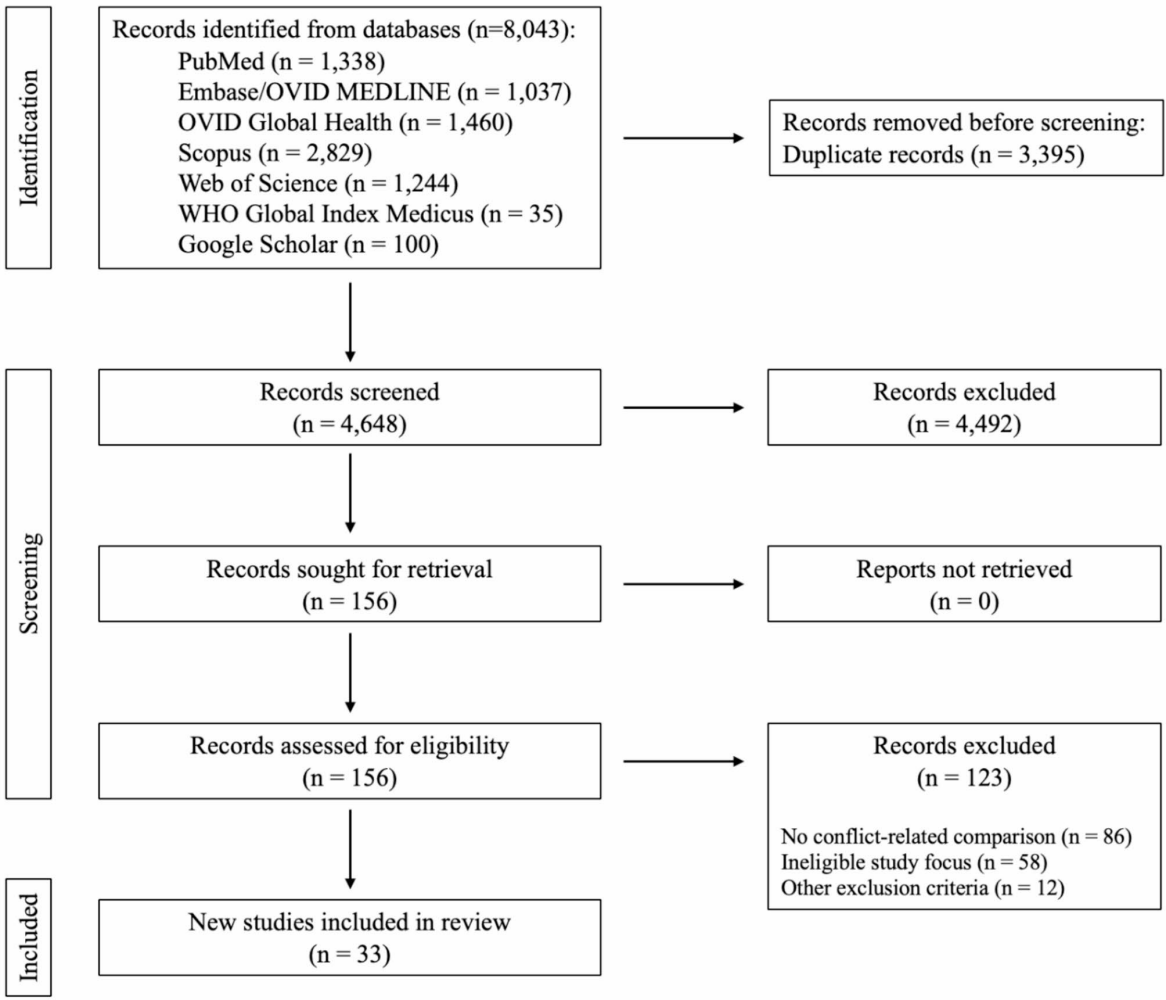
Preferred Reporting Items for Systematic Reviews and Meta-Analyses (PRISMA) flow diagram: publication selection process for systematic review on the impact of armed conflict on immunization coverage

A total of 8,043 citations were retrieved through the database search. After removing duplicates, 4,648 citations remained and were screened by title and abstract based on the study’s predefined eligibility criteria. Of these, 4,492 did not meet the inclusion criteria. We reviewed 156 full-text articles, of which 33 were eligible for inclusion (see Fig. 1). Of the 123 studies excluded, 86 (55%) did not include a conflict-related comparison, 58 (37%) had an ineligible study focus (e.g., internally displaced persons, healthcare workers, etc.), and 12 (8%) met other exclusion criteria (e.g., publication timeframe, systematic reviews, etc.). The characteristics of all included studies are presented in the additional file (Additional Table 1).

**Fig. 2:**
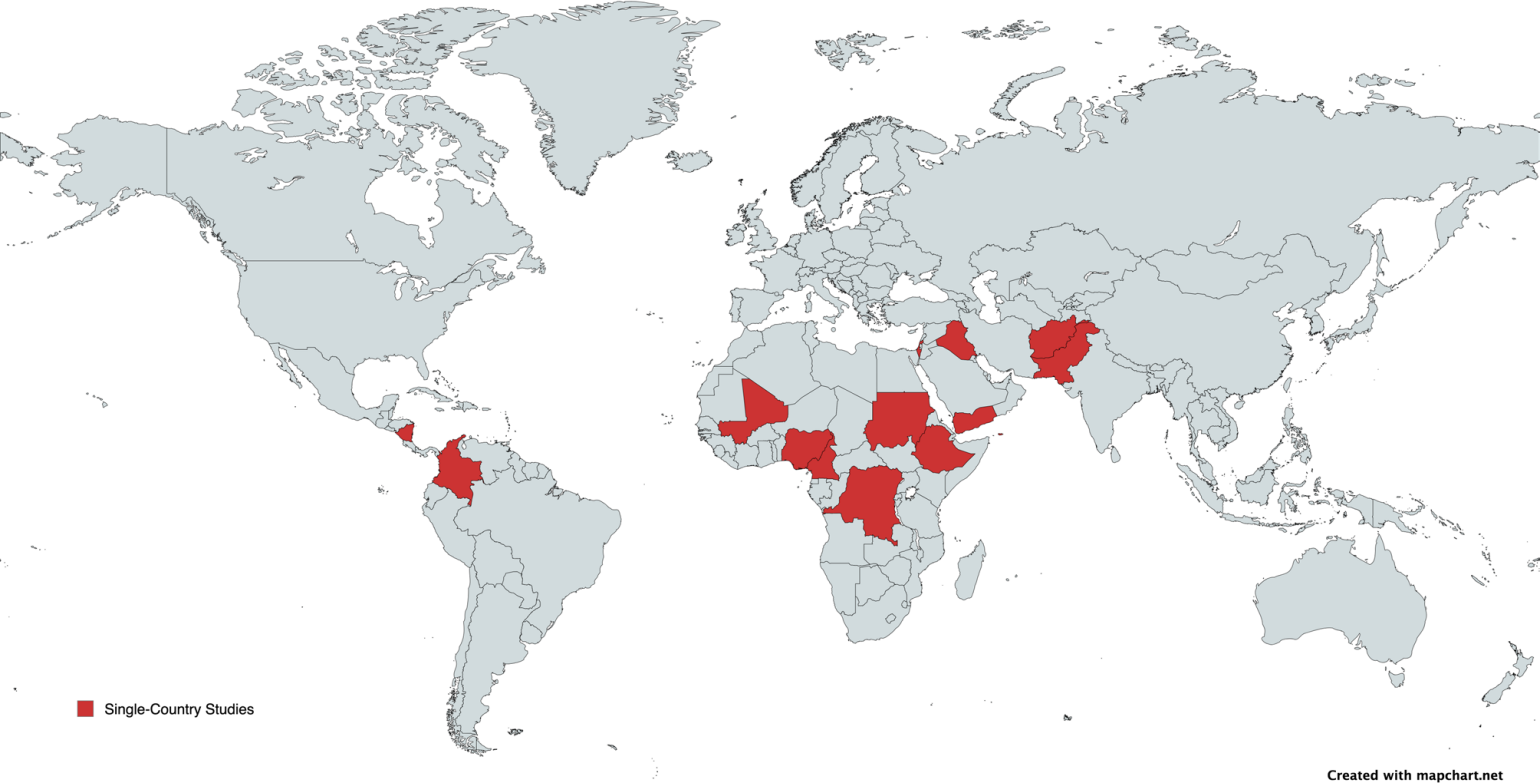
Single-Country Studies Included in the Systematic Review.*This map does not include countries which were included in multi-country studies. This map is licensed under a Creative Commons Attribution-ShareAlike 4.0 International License (CC BY-SA 4.0)

As shown in Table 1, most included studies (27, 82%) reported on individual countries, while 3 (9%) addressed regional contexts and another 3 (9%) presented multi-regional or global analyses. Among the 30 studies with an individual or regional focus, analyses were primarily concentrated in the Eastern Mediterranean (15, 45%), African (12, 36%), and Americas (3, 9%) regions, according to WHO regional classifications. As shown in Fig. 2, no studies explicitly focused on the European, South-East Asian, or Western Pacific regions. Nearly all studies (32, 97%) were published in English. Using the standardized JBI critical appraisal scale (0 = highest risk of bias; 8 = lowest risk of bias), the mean quality score was 6.6 (range 3–8). Descriptive and observational studies had a lower mean score (6.0; range 3–8) compared to quasi-experimental studies (7.4; range 6–8), indicating greater methodological rigor in the latter.

**Table 1:** Characteristics of the 33 Included Studies

Characteristic	N (%)
Publication year	
1985–1999 2000–2009 2010–2019 2020–2025	1 (3) 2 (6) 9 (27) 21 (64)
Geographic focus	
Individual Countries Regional Multi-Regional or Global	27 (82) 3 (9) 3 (9)
Geographic region	
AFR (African Region) AMR (Region of the Americas) SEAR (South-East Asian Region) EUR (European Region) EMR (Eastern Mediterranean Region) WPR (Western Pacific Region) Multiple	12 (36) 3 (9) 0 (0) 0 (0) 15 (45) 0 (0) 3 (9)
Language	
English Spanish	32 (97) 1 (3)
Study design	
Observational Quasi-Experimental Descriptive	16 (48) 15 (45) 2 (6)

AFR: African Region; AMR: Region of the Americas; SEAR: South-East Asian Region; EUR: European Region; EMR: Eastern Mediterranean Region; WPR: Western Pacific Reg

### Conflict exposure

There was no standardized metric for measuring the intensity, scale, or scope of armed conflict. The most common data source was peer-reviewed conflict datasets (13, 39%), followed by author-defined or inferred metrics (6, 18%) [17–22], governmental or institutional data (3, 9%) [23–25], NGO or research organization data (3, 9%) [26–28], primary data (3, 9%) [29–31], and primary aggregation of media or journalism information (1, 3%) [32]. Conflict data was not reported, unclear, or unused in four studies (12%) [33–36]. The Uppsala Conflict Data Program (UCDP) dataset was by far the most used source of conflict data, employed by 11 studies (33%). As shown in Table 2, there was substantial variation in how conflict intensity was measured across studies. Nearly half (15, 45%) used BRDs, while 8 (24%) relied on qualitative, subjective, or expert-rated measurements. Seven (21%) studies applied quantitative proximity or exposure-derived metrics, 4 (12%) used event-based metrics, 2 (6%) utilized quantitative victimization-specific metrics. Six (18%) studies either did not report their metric or did not use a specific measure of conflict intensity. Notably, 8 (24%) studies employed more than one type of measurement. To enable cross-study comparison, average annual BRDs were calculated for each included study, where possible, using UCDP data over the study’s observation period to approximate conflict intensity. Nearly half of the 33 studies (16, 48%) analyzed conflicts with between 1,001 and 5,000 annual BRDs [17–19, 21, 24, 25, 28–30, 32–34, 37–40], 5 (15%) studies analyzed conflicts with 101-1,000 BRDs [20, 22, 26, 35, 36], 4 (12%) analyzed conflicts with 5,001–10,000 BRDs [27, 41–43], and 1 (3%) analyzed a conflict with greater than 10,000 BRDs [31]. As many studies examined conflict at the sub-national rather than national level, these BRD categories should be interpreted as approximate reference ranges rather than definitive indicators of conflict intensity.

**Table 2:** Characteristics of conflict and immunization data and metrics in included studies

Data or Metric	N (%)
Source of conflict exposure data	
Peer-reviewed conflict dataset Author-defined or inferred metric Governmental or institutional data NGO or research organization data Primary data Aggregation of media or journalism Not reported, unclear, or unused	13 (39) 6 (18) 3 (9) 3 (9) 3 (9) 1 (3) 4 (12)
Measures of conflict intensity*	
Quantitative: BRD-based Quantitative: Event-based Quantitative: Proximity/Exposure Quantitative: Victimization-specific Qualitative/Subjective or Expert-rated No metric specified or used	15 (45) 4 (12) 7 (21) 2 (6) 8 (24) 6 (18)
Conflict intensity (based on average annual BRDs at the national level during study period per UCDP)	
1-100 BRDs 101-1,000 BRDs 1,001–5,000 BRDs 5,001–10,000 BRDs >10,000 BRDs NA (multi-country; conflict began pre-1985)	0 (0) 5 (15) 16 (48) 4 (12) 1 (3) 7 (21)
Sources of immunization data**	
Household Surveys: International Standardized Household Surveys: Primary/Study-Specific Administrative Data: Routine Health System Administrative Data: Aggregated Reports	19 (58) 3 (9) 8 (24) 4 (12)
Vaccines assessed	
DTP (DTP1, DTP2, DTP3)*** BCG (Tuberculosis, TB) Pentavalent/Penta-3 (DTP-HepB-Hib) Polio (IPV or OPV, polio 1/2/3)**** Measles, Mumps, Rubella (MR, MMR) Pneumococcal conjugate (PCV, PCV-13) Rotavirus or Rotarix-2 (Rotavirus, Rotarix, RotaTeq) Tetanus (TT)***** HiB Hepatitis B (HBV, HepB) Tuberculosis (TB) Unclear	17 (52) 20 (61) 7 (21) 13 (39) 17 (52) 4 (12) 2 (6) 3 (9) 2 (6) 2 (6) 1 (3) 3 (9)
Immunization outcomes	
Coverage rate Dropout rate Timeliness of vaccination Campaign reach and access Ever vaccinated/vaccination uptake Composite indicators	22 (67) 3 (9) 2 (6) 1 (3) 5 (15) 2 (6)
Target populations*	
Children Women of reproductive age	31 (94) 3 (9)

*Eight (24%) studies used multiple types of measurements and one (3%) study focused on both children and women of reproductive age. **One (3%) study included multiple sources of immunization data. ***Does not include DTP when pentavalent or Penta-3 vaccine explicitly measured. ****IPV and OPV are combined because some papers did not specify which polio vaccine was measured. *****Does not include tetanus when pentavalent, DTP, or Penta-3 vaccines were explicitly measured

BCG: Bacille Calmette-Guérin; BRD: Battle-Related Deaths; DTP: Diphtheria, Pertussis, Tetanus; HepB: Hepatitis B; HBV: Hepatitis B Virus; HiB: Haemophilus influenzae type B; IPV: Inactivated Poliovirus Vaccine; MMR: Measles, Mumps, Rubella; MR: Measles, Rubella; NA: Not Applicable; NGO: Non-Governmental Organization; OPV: Oral Poliovirus Vaccine; PCV: Pneumococcal Conjugate Vaccine; PCG-13: 13-valent Pneumococcal Conjugate Vaccine; TB: Tuberculosis; TT: Tetanus Toxoid; UCDP: Uppsala Conflict Data Program

### Immunization data

As shown in Table 2, the primary source of immunization data across included studies was international standardized household surveys (19, 58%), including the Demographic and Health Surveys (DHS) and Multiple Indicator Cluster Surveys (MICS). Routine administrative data from national or subnational health systems (e.g., EPI and DHIS2) were used in 8 (24%) studies [22, 25, 32–36, 39]. Other sources included aggregated administrative reports compiled by ministries of health or international agencies (4, 12%) [17, 30, 44, 45] and study-specific household surveys (3, 9%) [23, 29, 31]. A broad range of vaccines was assessed, most commonly BCG (20, 61%), DTP (17, 52%), and measles-containing vaccines (17, 52%). Polio (13, 39%) and pentavalent vaccines (7, 21%) were also frequently examined, the latter of which includes DTP. Coverage rate was the most used immunization outcome (22, 67%), followed by vaccination uptake (5, 15%) [27, 37, 38, 40, 43], dropout rate (3, 9%) [22, 31, 36], timeliness (2, 6%) [21, 26], composite indicators (2, 6%) [28, 45], and campaign reach or access (1, 3%) [32]. Two (6%) studies reported multiple outcomes [22, 36]. Most studies (31, 94%) focused on child vaccination; three (9%) assessed coverage in women of reproductive age [28, 42, 46], and one (3%) examined both populations [42].

**Table 3:** Characteristics and main findings of each included study

Short citation	Publication year	Country/Region	Language	Study design	Unit of analysis	Main effect size	Bias (JBI)
Akseer et al. [41]	2019	Afghanistan	English	Quasi-Experimental	District	Moderate-to-severe conflict associated with lower vaccine coverage: BCG − 3.8 pp/year (*p* = 0.002), measles − 2.9 pp/year (*p* = 0.01), DTP3 − 3.0 pp/year (*p* < 0.001) compared to minimal conflict.	8
Al-Samhari et al. [33]	2023	Yemen	English	Observational	Individual	Annual vaccination coverage declined: APC − 1.92 by 2018.	5
Amberg et al. [47]	2023	Sub-Saharan Africa	English	Quasi-Experimental	Individual	Exposure to nearby armed conflict was associated with a −2.5pp reduction in timely receipt of all basic vaccinations (95% CI −3.1 to −1.9). Longer conflicts (≥ 5 years) and higher intensity (≥ 1000 deaths) showed larger reductions of −5.9pp (95% CI −7.2 to −4.6) and − 9.9pp (95% CI −13.3 to −6.6), respectively.	7
Batarfi et al. [17]	2024	Yemen	English	Descriptive	District	Between 2015 and 2020, vaccination coverage in Coastal Hadhramaut declined across multiple vaccines. BCG fell by up to 15%, IPV by 14%, and MR coverage dropped intermittently, with only 4 of 12 districts achieving ≥ 80% MR1 coverage in 2020. Rotavirus and pentavalent vaccines declined by up to 9%, with similar decreases in OPV and PCV, reflecting broad disruptions from conflict and COVID-19.	3
Broholm et al. [23]	1989	Nicaragua	English	Observational	Household/child-level data aggregated by town	Children in the conflict-affected town of Acoyapa were significantly less likely to be fully vaccinated than those in the non-conflict town of El Ostional (OR 0.17, 95% CI 0.07–0.39). Immunization card possession was also markedly lower in Acoyapa (OR 0.23, 95% CI 0.09–0.57).	5
Cetorelli, Valeria [21]	2015	Iraq	English	Quasi-Experimental	Individual (child)	War-exposed children 21.5pp less likely to receive neonatal polio vaccine (95% CI − 34.1 to − 8.9).	7
Chepkurui et al. [45]	2021	WHO Africa Region (13 beneficiary countries of PHE mitigation funds from the African Public Health Emergency Fund)	English	Descriptive	National	Greater frequency of armed conflict and emergencies significantly associated with failure to reach 90% DTP3 coverage.	4
El Bcheraoui et al. [18]	2018	Yemen	English	Observational	Governate	Coverage declined by 16.2% for DTP3, 17.9% for measles vaccine, 13.1% for pneumococcal vaccine, and 14.3% for polio3.	8
Goli et al. [48]	2022	52 developing countries	English	Quasi-Experimental	Individual	Exposure to conflict associated with reductions in full vaccination: −0.4% per conflict year; −2.3 to − 2.8% for high mortality conflicts.	7
Grossman et al. [37]	2019	Pakistan	English	Quasi-Experimental	Individual/household	A one SD increase in attack intensity before birth was associated with 2–7 fewer children per 1,000 receiving the tuberculosis vaccine. Exposure during the final two to three months of pregnancy resulted in 7–8 fewer children per 1,000 being vaccinated.	7
Haddison et al. [36]	2020	Cameroon	English	Observational	Regional	DTP3 dropped from 90% (2017) to 55% (2018); BCG from 87% (2017) to 54% (2018).	4
Jawad et al. [44]	2021	Worldwide	English	Observational	National	Armed conflict was associated with a 2.6% (95% CI 0.2–5.0) reduction in measles vaccine coverage, while wars were linked to 4.9% (95% CI 1.5–8.3) and 7.3% (95% CI 2.7–11.8) reductions in DTP and measles coverage, respectively. These effects persisted for up to three years (DTP) and two years (measles) following war onset.	8
Kreif et al. [24]	2022	Colombia	English	Observational	Household	No statistically significant association between conflict and vaccination coverage.	8
Leone et al. [26]	2019	Occupied Palestinian Territory (oPt)	English	Observational	Individual	Conflict intensity negatively associated with OPV: β=−0.223 (*p* < 0.001); and DTP: β=−0.227 (*p* < 0.001).	8
Malembaka et al. [39]	2021	Kivu, DR Congo	English	Quasi-Experimental	Health zone level	Under the most inclusive conflict threshold, the estimated effect on DTP3 coverage was 1.35 (95% CI − 1.67 to 4.37), indicating a non-significant positive association between severe conflict and DTP3 coverage. No statistically significant results were reported at any other conflict level.	7
Mashal et al. [30]	2007	Afghanistan	English	Observational	District	Compared with highly insecure regions, occasionally insecure areas had significantly higher odds of achieving ≥ 80% vaccination coverage for BCG (OR 3.13, 95% CI 1.56–6.31, *p* < 0.001), DTP3 (OR 12.16, 95% CI 3.40–43.55, *p* < 0.001), OPV3 (OR 2.99, 95% CI 1.21–7.42, *p* = 0.02), and measles (OR 10.31, 95% CI 2.71–39.23, *p* < 0.001). In insecure regions, odds were higher for DTP3 (OR 4.17, 95% CI 1.06–16.38, *p* = 0.04) and OPV3 (OR 3.09, 95% CI 1.03–9.29, *p* = 0.04), but not significant for BCG or measles.	8
Masset, Edoardo [20]	2022	Mali	English	Quasi-Experimental	Region	In a comparison between conflict-affected North and non-conflict South, vaccination coverage was 8% lower in the North (SE = 0.041).	8
Mezen et al. [31]	2023	Ethiopia	English	Observational	Household	44% of children initiating vaccination before war dropped out; conflict duration inversely associated with dropout: OR 1.75 (95% CI 1.11–2.77) for 1-month exposure; OR 0.51 (95% CI 0.28–0.93) for 5-month exposure.	6
Mirzazada et al. [42]	2020	Afghanistan	English	Observational	Province (for conflict classification); Individuals (for outcomes)	Severe conflict was associated with a 74% reduction in the odds of DTP3 vaccination (OR: 0.26; 95% CI: 0.20–0.33; *p* < 0.0001), a nearly threefold increase in BCG vaccination (OR: 2.80; 95% CI: 2.09–3.76; *p* < 0.0001), and no statistically significant change in measles vaccination (OR: 1.36; 95% CI: 0.95–1.94; *p* = 0.096)	8
Mohamed et al. [28]	2022	Sudan	English	Observational	Individual	Women living in low-intensity armed conflict areas had significantly higher odds of receiving adequate tetanus vaccination compared to those in high-intensity areas (AOR: 1.34; 95% CI: 1.14–1.57).	8
Naufal et al. [27]	2020	Iraq	English	Quasi-Experimental	Individual child	Probit coefficients for high-conflict exposure were 0.178 for any vaccine (*p* < 0.01), 0.123 for BCG (*p* < 0.05), and 0.090 for measles/MMR (*p* < 0.05), indicating significant positive associations; no significant effects were observed for polio, Hep B, DTP, or pentavalent vaccines.	8
Ojeleke et al. [38]	2022	Nigeria	English	Quasi-Experimental	Individual-level nested within clusters	Child immunization odds were reduced by 14–19% within 5 km (OR 0.81–0.86) and 18–38% within 10 km (OR 0.62–0.82) of conflict, paralleling declines seen in antenatal care and facility births.	8
Omer et al. [19]	2014	Sudan	English	Observational	State	Children in conflict-affected states had 4.5pp lower full vaccination rates.	4
Østby et al. [49]	2021	Sub-Saharan Africa (15 countries)	English	Quasi-Experimental	Household	Minor conflict associated with slight increase in immunization (+ 0.002); major conflict associated with modest decrease (− 0.001); 100 BRD increase associated with − 0.004 probability of full immunization.	7
Ruiz, Gladys Cecilia Ghisays [25]	2003	Colombia	Spanish	Observational	Municipality	Municipalities where the armed conflict was present had 7% less coverage than municipalities where the conflict was not present.	5
Saidu et al. [35]	2021	Cameroon	English	Observational	Health district	BCG and DTP3 coverage declined by 22pp and 42pp, respectively (2016–19); similar trends for PCV13, IPV, and Rotavirus vaccines.	4
Sato, Ryoko [40]	2019	Nigeria	English	Observational	Household	Conflict events during or shortly after childbirth reduced odds of any vaccination by 41.9–48.0%, and conflict events within two months of childbirth were associated with up to 59% lower likelihood for BCG and 56.1% for DTP1. No significant effect was observed when conflict occurred three months after birth.	8
Sato, Ryoko [43]	2021	Nigeria	English	Quasi-Experimental	Individual child (clustered by geographic location)	In Boko Haram–affected areas (Yobe, Adamawa, and Borno), conflict exposure was associated with a 35% reduction in the odds of a child ever being vaccinated (OR 0.65, 95% CI 0.44–0.97, *p* < 0.01), with stronger negative effects observed for DTP3/Penta3 uptake (OR 0.61, 95% CI 0.41–0.91, *p* < 0.01). The effect on BCG vaccination was smaller and not statistically significant (OR 0.97, 95% CI 0.66–1.43). Across Nigeria overall, conflict exposure was linked to a 26% increase in odds of any vaccination (OR 1.26, 95% CI 1.01–1.56, *p* < 0.05) and a 52% increase in BCG vaccination (OR 1.52, 95% CI 1.24–1.86, *p* < 0.001), with no significant effect on DTP3/Penta3 uptake (OR 1.05, 95% CI 0.90–1.23).	8
Schaub et al. [29]	2025	Nigeria	English	Quasi-Experimental	Individual (caregiver–child dyads)	The odds of full immunization were reduced by 57% (OR 0.43, 95% CI 0.32–0.57).	7
Tewantsa et al. [22]	2024	Cameroon	English	Quasi-Experimental	District	In the North West, PENTA3 coverage decreased by 7% (Z = 3.40, *p* < 0.001) and RR1 by 8% (Z = 4.14, *p* < 0.001). In the South West, coverage declined by 22% for BCG, 33% for PENTA3, and 36% for RR1 (all *p* < 0.001).	6
Torbosh et al. [34]	2019	Yemen	English	Observational	Governate/national	National measles coverage declined from 75–66% (2014–15); Penta-3 from 88–84%; BCG from 73–49%, especially in conflict-affected governorates.	4
Verma et al. [32]	2018	Pakistan	English	Quasi-Experimental	District-level vaccination campaign; district-month for polio incidence	Vaccination coverage was 5.3% lower (95% CI 5.2–5.3) in high-insecurity campaigns compared with secure campaigns.	8
Zhang et al. [46]	2023	Chad, CAR, DRC, and Iraq	English	Quasi-Experimental	Individual	Armed conflict positively associated with maternal tetanus vaccination: β = 0.055 (95% CI 0.004–0.106; *p* < 0.05).	8

AOR: Adjusted Odds Ratio; BCG: Bacillus-Calmette-Guérin; BRD: Battle-Related Deaths; CAR: Central African Republic; CI: Confidence Interval; COVID-19: Coronavirus Disease 2019; DHS: Demographic and Health Surveys; DTP: Diphtheria, Pertussis, Tetanus; DTP1: First dose of DTP vaccine; DTP3: Third dose of DTP vaccine; DRC: Democratic Republic of the Congo; EPI: Expanded Programme on Immunization; HBV: Hepatitis B Virus; Hib: Haemophilus influenzae type b; IPV: Inactivated Polio Vaccine; JBI: Joanna Briggs Institute; MICS: Multiple Indicator Cluster Surveys; MMR: Measles, Mumps, Rubella; MR: Measles, Rubella; MR1: First dose of Measles, Rubella vaccine; NA: Not Applicable; OPV: Oral Polio Vaccine; OPV3: Third dose of Oral Polio Vaccine; OR: Odds Ratio; oPt: Occupied Palestinian Territory; PCV: Pneumococcal Conjugate Vaccine; PCV13: 13-valent Pneumococcal Conjugate Vaccine; PENTA3: Third dose of Pentavalent Vaccine; pp: Percentage Points; RR1: First dose of Rotavirus vaccine; SD: Standard Deviation; SE: Standard Error; TB: Tuberculosis; UCDP: Uppsala Conflict Data Program; WHO: World Health Organization

### Study design and outcomes

Of the 33 included studies, 16 (48%) employed observational designs; 15 (45%) were quasi-experimental; and two (6%) were descriptive (see Table 3). As presented in Tables 4 and 85% (28 of 33) of included studies reported a decline in immunization coverage during armed conflict, including 94% (15 of 16) of observational studies [18, 19, 23, 25, 26, 28, 30, 31, 33–36, 40, 42, 44], 73% (11 of 15) of quasi-experimental studies [20–22, 29, 32, 37, 38, 41, 43, 47, 48], and all descriptive studies (2 of 2) [17, 45]. Across all study estimates reporting absolute percentage changes in vaccination coverage, the average change was − 11.4% points [17–21, 25, 33, 35, 36, 39, 41, 44, 47, 48], with effects ranging from a modest increase of + 1.35% points [39] to a decline of −42% points [35]. As shown in Table 3, individual-level analyses were most common (10 of 33, 30%), followed by subnational administrative (9 of 33, 27%), community or cluster (6 of 33, 18%), household (5 of 33, 15%), and national-level analyses (3 of 33, 9%). Reductions in vaccination coverage were observed across nearly all units of analysis, with consistent declines reported in all (9 of 9, 100%) administrative-level studies [17–20, 22, 30, 32, 36, 41] and in 80% (8 of 10) of individual-level studies [21, 26, 28, 29, 33, 42, 47, 48] (see Tables 3 and 4). Two quasi-experimental studies using individual-level data reported increases in coverage [27, 46], suggesting possible local variability in service delivery potentially due to targeted interventions or measurement bias. Outcomes also varied by conflict type: all studies on civil war (7 of 7, 100%) [17–19, 28, 31, 33, 34] and military occupation (1 of 1, 100%) [26] reported declines in coverage, as did most studies on insurgency (13 of 16, 81%) [20–22, 25, 29, 30, 35, 36, 38, 40–43] and all studies examining combined insurgency and terrorism (2 of 2, 100%) [32, 37]. As shown in Table 4, stratification by conflict intensity at the national level, measured by average annual BRDs across the study period, showed that 88% (14 of 16) of studies [17–19, 21, 25, 28–30, 32–34, 37, 38, 40] on conflicts with 1,001–5,000 deaths/year reported negative effects, as did all studies on conflicts with fewer than 1,000 deaths/year (5 of 5) [20, 22, 26, 35, 36], most on conflict with 5,001–10,000 deaths/year (3 of 4) [40–42], and the sole study on conflict with more than 10,000 deaths/year [31]. Among studies with unclassified intensity, 71% (5 of 7) also found decreased coverage [23, 44, 45, 47, 48]. 

**Table 4:** Study design characteristics and outcomes

Characteristic	Study results (N, %)				
	Decrease	Non-monotonic	No change	Increase	Total
Main Effect Direction	28 (85)	1 (3)	1 (3)	3 (9)	33
Study Design					
Observational Quasi-Experimental Descriptive	15 11 2	0 1 0	1 0 0	0 3 0	16 (48) 15 (45) 2 (6)
Unit of Analysis					
Individual Household Community/Cluster Administrative Subnational National	8 3 5 9 3	0 1 0 0 0	0 1 0 0 0	2 0 1 0 0	10 (30) 5 (15) 6 (18) 9 (27) 3 (9)
Type of Armed Conflict					
Low-Intensity Military Occupation Insurgency Insurgency/Terrorism Civil War NA (multi-country study)	1 1 13 2 7 4	0 0 0 0 0 1	0 0 1 0 0 0	0 0 2 0 0 1	1 (3) 1 (3) 16 (48) 2 (6) 7 (21) 6 (18)
Conflict intensity*					
1-100 BRDs 101-1,000 BRDs 1,001–5,000 BRDs 5,001–10,000 BRDs >10,000 BRDs NA (multi-country; pre-1985)	0 5 14 3 1 5	0 0 0 0 0 1	0 0 1 0 0 0	0 0 1 1 0 1	0 (0) 5 (15) 16 (48) 4 (12) 1 (3) 7 (21)

*Based on average annual BRDs at the national level during study period (per UCDP)

BRD: Battle-Related Deaths; NA: Not Applicable

Several studies included in this review demonstrate that armed conflict substantially disrupts immunization coverage among children across diverse

conflict-affected settings. In Afghanistan, conflict intensity was linked to annual declines in vaccine coverage, including BCG (− 3.8% points per year), measles (− 2.9 pp/year), and DTP3 (− 3.0 pp/year), compared to areas experiencing minimal conflict [41]. Similar patterns were observed in Yemen, where measles coverage declined from 75–66%, and BCG coverage dropped sharply from 73–49% during conflict periods [34], reflecting the broad disruptions to vaccination services caused by conflict [17]. In sub-Saharan Africa, multi-country analyses revealed that prolonged and intense conflicts were associated with reductions in timely receipt of basic vaccines by up to 9.9% points [47], while national-level studies highlighted failure to reach key thresholds such as 90% DTP3 coverage in countries with frequent conflicts [45]. Similarly, in Nigeria, proximity to conflict within 10 km reduced odds of child immunization by 14–38% [38], with Boko Haram insurgency zones experiencing a 35% reduction in vaccination odds for key vaccines [43].

The timing and duration of conflict also influence immunization outcomes. In northeastern Nigeria, conflict events during or shortly after childbirth were associated with 42–59% lower odds of vaccination, with effects diminishing as the interval from conflict events increased beyond two months from birth [40]. Conversely, in Ethiopia, children initiating vaccination before war experienced high dropout rates, and conflict duration showed an inverse association with dropout odds, suggesting complex dynamics between exposure and service disruption [31]. To this end, some contexts reveal nuanced or even counterintuitive findings; armed conflict was associated with a modest increase in maternal tetanus vaccination in Chad, Central African Republic (CAR), Democratic Republic of the Congo (DRC), and Iraq [46], and in parts of Nigeria, conflict exposure corresponded with increased odds of any vaccination and BCG coverage outside Boko Haram areas [43]. These disparities may reflect differences in conflict characteristics, health systems resilience, or humanitarian responses. Some studies also explored whether individual- or household-level factors modulate conflict’s effects on vaccination rates. Although evidence on gender differences was limited and mixed [26, 47, 49], studies consistently showed that higher household wealth [21, 26, 28, 40, 47, 49] and parental education [26, 28, 38, 40, 47, 49] were associated with increased likelihood of childhood vaccination in conflict-affected settings.

Studies conducted at the individual (n = 10) and household (n = 5) levels demonstrated greater heterogeneity in the direction and magnitude of effects, with several reporting modest reductions in coverage or null findings. Notably, two individual-level studies reported increases in vaccination coverage, both employing quasi-experimental designs [27, 46]. In contrast, all studies at the administrative subnational level (n = 9) reported declines in vaccination rates [17–20, 22, 30, 32, 36, 41], suggesting more consistent negative effects at aggregated levels of analysis. Similarly, all three national-level studies [34, 44, 45] and five of six cluster-level studies [23, 25, 35, 38, 43] also identified reductions in coverage. These findings suggest that studies using higher-level administrative or aggregated data may more consistently capture systemic disruptions to immunization services, whereas individual- and household-level studies may reflect greater local variability in service delivery, access, or data quality.

Overall, robust evidence from quasi-experimental and observational studies in multiple countries—including Afghanistan, Iraq, Cameroon, Yemen—and regions like sub-Saharan Africa consistently documents substantial declines in full vaccination coverage and increased dropout rates attributable to armed conflict. In some settings, war-exposed children were over 20% points less likely to receive key vaccines such as polio and DTP [21, 26]. Moreover, the adverse effects of conflict on vaccination coverage often persist for several years post-conflict [44], highlighting the long-term impact of armed violence on routine immunization programs and the broader implications for child health. These findings underscore the critical need for targeted, context-specific strategies to maintain immunization services in conflict-affected settings and to strengthen resilience within fragile health systems.

## Discussion

This systematic review finds that armed conflict is a major disruptor of routine immunization services, with 85% of included studies reporting reduced vaccination coverage across diverse settings. Conflicts involving civil war or military occupation were especially likely to result in substantial reductions in coverage for critical vaccines such as DTP, BCG, and polio. The negative impact of conflict on immunization appeared to intensify with greater conflict intensity, particularly when BRDs exceeded 1,000 annually. These findings underscore the vulnerability of health systems to armed conflict and highlight the importance of understanding both the type and severity of conflict when evaluating its impact on health service delivery.

There was considerable heterogeneity in study design and units of analysis, which appeared to influence both the magnitude and direction of observed conflict effects. Studies using administrative or national-level data more consistently identified substantial declines in coverage, while those using household or individual-level data reported more mixed results. This discrepancy likely reflects both methodological and structural factors. Aggregated data may more accurately capture health system-wide service interruptions, particularly in areas where vaccination services are suspended across entire provinces or districts due to safety concerns. Localized studies, especially those using quasi-experimental methods, may overemphasize granular variability due to individual conflict events and miss broader trends since their limited scope makes it difficult to capture systemic or structural disruptions that unfold across wider geographic areas. The impact of conflict may further be underestimated in individual and household-level studies, which are often limited to populations that remain accessible to survey enumerators, and by extension to health workers, thereby potentially diluting observed effects. For these reasons, granular analyses at the individual, household, community level may obscure the causal impact of conflict on vaccination coverage in high-conflict settings and may be more appropriate for assessing effects in low-to-medium intensity conflict environments.

Conflict rarely occurs in isolation. Overlapping crises, such as the COVID-19 pandemic, climate-related disasters, and economic instability, compound the strain on health systems already weakened by conflict. For instance, the COVID-19 pandemic may have prolonged or deepened conflict-related declines in vaccination coverage by further restricting health worker access to affected areas, diverting resources to outbreak response, constraining international vaccine supply chains, and reducing health worker availability. While several studies included in this review were conducted during such compound emergencies, none systematically account for these concurrent stressors. The absence of multi-crisis analytical frameworks limits our ability to disentangle the specific effects of conflict on vaccination disruptions. Future research should integrate broader contextual factors that contribute to health system collapse and disrupt vaccine delivery. Similarly, factors such as health systems resilience, capacity of national institutions, and adherence to global policy frameworks (e.g., Global Health Security) can affect a country’s ability to sustain health services during emergencies and health system shocks [50, 51]. These structural characteristics were rarely accounted in studies, yet they are critical for understanding variation in outcomes and designing effective interventions and policy responses.

Temporal and spatial dynamics of disruption in conflict-affected settings remain poorly characterized. Few studies employed longitudinal or geospatial analyses capable of capturing changes in vaccination coverage over time or across subnational areas despite the fact that conflict occurrence and intensity fluctuate temporally and geographically. Such approaches are essential for identifying the lagged effects between conflict onset and service disruption, and for distinguishing between direct and spillover effects, particularly in regions bordering active conflict zones. Compounding this issue, population displacement, a central mechanism through which conflict shapes health outcomes, is insufficiently integrated to existing analyses. Internally displaced persons (IDPs), war refugees, and cross-border populations are frequently excluded from household surveys and administrative datasets, creating significant blind spots in estimating the true causal impact of conflict on immunization rates. Their exclusion likely contributes to a systematic underestimation of the public health burden, and it is unclear to what extent forcible displacement acts as a driver of vaccination coverage declines independent of conflict or can be used to forecast vaccination coverage declines. Digital innovations, such as mobile health platforms, electronic registries, and satellite-enabled survey techniques, can potentially strengthen data collection and surveillance in hard-to-reach or unstable regions. In addition, few studies incorporated the impact of humanitarian ceasefire agreements that explicitly aim to facilitate immunization, despite historical precedents (e.g., “Days of Tranquility” for polio). Such geopolitical determinants can shape both access to vaccination and data availability and should be more consistently examined in future evaluations.

A key limitation across the existing body of evidence is the marked heterogeneity in methods, metrics, and data sources used to assess both conflict exposure and immunization outcomes. The absence of standardized definitions of conflict exposure—ranging from national-level conflict listings to geocoded violence data—hampers comparisons and limits the generalizability of findings across contexts. Similarly, vaccination indicators varied widely, with studies employing diverse metrics such as binary uptake metrics, dropout rates, and composite indices. This variation poses significant challenges for synthesis and interpretations. The field would greatly benefit from standardized frameworks for measuring both conflict exposure and disruption to vaccination services, which would enhance comparability and facilitate meta-analytic approaches. Such frameworks, particularly for descriptive and observational studies, may also help address quality concerns identified using JBI tools: descriptive and observational studies had a lower mean score (6.0; range 3–8) compared to quasi-experimental studies (7.4; range 6–8), suggesting that their results may be less robust. Some of the largest coverage declines were reported in descriptive studies with low JBI scores. While this raises concerns about the reliability of these estimates, many of these lower-scoring studies focused on point estimates of vaccination coverage before and during conflict. As such, they may still provide timely and relevant estimates and capture abrupt declines in coverage without being influenced by extrapolation or modeling assumptions that might dilute the observed effect. Nevertheless, the lower methodological rigor highlights the need for additional robustness checks and adoption of a standardized measurement framework to strengthen confidence in their results. Finally, very few studies attempted to quantify the downstream health burden attributable to missed vaccinations using burden of disease metrics, such as Years of Life Lost (YLLs). While measuring declines in reduced coverage is a critical first step, it is insufficient without translating these service disruptions into health impact estimates to prioritize interventions, inform policy discussions, and guide resource allocation in conflict-affected settings.

Taken together, these findings affirm that armed conflict poses a severe and multifaceted threat to immunization programs. To support effective intervention and policymaking, future research should prioritize the use of standardized conflict exposure metrics, incorporate spatial and temporal analytic approaches, and generate disaggregated data, particularly on displaced populations and those facing gender-related vulnerabilities. Methodological and technological advancements will be essential to safeguarding immunization efforts in an era increasingly shaped by overlapping global crises.

## Conclusions

Armed conflict consistently disrupts routine immunization services, though the magnitude of effects varies by conflict type, intensity, and analytic approach. To more accurately quantify and address these impacts, future research should adopt standardized methodologies, account for displaced populations, and estimate downstream health burdens. Strengthening this evidence base is critical to informing targeted interventions and safeguarding immunization programs in fragile and conflict-affected settings.

## Supplementary Information

Supplementary Material 1.

Supplementary Material 2.

## Data Availability

No datasets were generated or analysed during the current study.

## Abbreviations

AFR: African region
AOR: Adjusted odds ratio
AMR: Region of the Americas
BCG: Bacillus-Calmette-Guérin
BRD: Battle-related deaths
CAR: Central African Republic
CI: Confidence interval
COVID-19: Coronavirus disease 2019
DALY: Disability-adjusted life year
DHS: Demographic and health surveys
DHIS2: District health information system 2
DTP: Diphtheria, pertussis, tetanus
DTP1: First dose of DTP vaccine
DTP3: Third dose of DTP vaccine
DRC: Democratic Republic of the Congo
EMR: Eastern Mediterranean region
EPI: Expanded programme on immunization
EUR: European region
HBV: Hepatitis B virus
HepB: Hepatitis B
HiB: Haemophilus influenzae type B
IPV: Inactivated poliovirus vaccine
JBI: Joanna Briggs institute
MICS: Multiple indicator cluster surveys
MMR: Measles, Mumps, Rubella
MR: Measles, Rubella
MR1: First dose of Measles, Rubella vaccine
NA: Not applicable
NGO: Non-governmental organization
OPV: Oral poliovirus vaccine
OPV3: Third dose of Oral poliovirus vaccine
OR: Odds ratio
oPt: Occupied Palestinian territory
PCV: Pneumococcal conjugate vaccine
PCV13: 13-valent pneumococcal conjugate vaccine
PENTA3: Third dose of pentavalent vaccine
pp: Percentage points
PRISMA: Preferred reporting Items for systematic reviews and meta-analyses
PROSPERO: International prospective register of systematic reviews
RR1: First dose of rotavirus vaccine
SD: Standard deviation
SE: Standard error
SEAR: South-East Asian region
TB: Tuberculosis
TT: Tetanus toxoid
UCDP: Uppsala conflict data program
WHO: World Health Organization
WPR: Western Pacific region
YLL: Years of life lost
