# Supplementary material for: The impact of armed conflict on vaccination coverage: a systematic review of empirical evidence from 1985 to 2025
Source: Confl Health. 2025 Oct 14;19:71. doi: 10.1186/s13031-025-00708-7 (PMC12519846; doi:10.1186/s13031-025-00708-7)
Supplement: Supplementary file 1 — Supplementary Material 1. [file 13031_2025_708_MOESM1_ESM.docx]

Additional Table 1: Database Search Strategy for Systematic Review on Armed Conflict and Immunization Coverage

| **Database** | **Time Coverage** | **Search Query** | **Results (N)** |
| --- | --- | --- | --- |
| **XPUBMED** | Not specified | ("armed conflict" OR "violent conflict" OR "civil war" OR "war" OR "conflict-related displacement" OR "conflict areas" OR "conflict zones" OR "displaced populations" OR "post-conflict areas") AND ("vaccination rates" OR "immunization rates" OR "vaccination coverage" OR "vaccine access" OR "vaccine-preventable diseases" OR "vaccination" OR "immunization" OR "vaccine uptake" OR "vaccine coverage" OR “vaccines” OR “immunizations”) | 1,338 |
| **Embase/OVID MEDLINE®** | 1985 to January 2025 | Same as above | 1,037 |
| **OVID Global Health** | 1985 to 2025 Week 1 | Same as above | 1,460 |
| **Scopus** | Not specified | TITLE-ABS-KEY("armed conflict" OR "violent conflict" OR "civil war" OR "war" OR "conflict-related displacement" OR "conflict areas" OR "conflict zones" OR "displaced populations" OR "post-conflict areas") AND TITLE-ABS-KEY("vaccination rates" OR "immunization rates" OR "vaccination coverage" OR "vaccine access" OR "vaccine-preventable diseases" OR "vaccination" OR "immunization" OR "vaccine uptake" OR "vaccine coverage" OR “vaccines” OR “immunizations”) | 2,829 |
| **Web of Science Core Collection** | Not specified | Same as XPUBMED query above | 1,244 |
| **WHO Global Index Medicus** | Not specified | ("armed conflict" OR "violent conflict" OR "civil war" OR "war" OR "conflict-related displacement" OR "conflict areas" OR "conflict zones" OR "displaced populations" OR "post-conflict areas") AND ("vaccination rates" OR "immunization rates" OR "vaccination coverage" OR "vaccine access" OR "vaccine-preventable diseases" OR "vaccination" OR "immunization" OR "vaccine uptake" OR "vaccine coverage") | 35 |
| **Google Scholar** | First 10 pages only | ("war" OR "armed conflict" OR "violent conflict" OR "political instability" OR "displacement" OR "post-conflict recovery" OR "genocide" OR "terrorism" OR "military intervention" OR "conflict zones") AND ("vaccination" OR "vaccine" OR "immunization" OR "vaccination coverage" OR "immunization rates") AND ("observational study" OR "cross-sectional study" OR "cohort study" OR "case-control study" OR "qualitative study" OR "mixed-methods" OR "evaluation study") | ~100 reviewed |

*Search criteria were selected in accordance with the following PICO (Population, Intervention/Exposure, Comparison, Outcome) framework: Population (P): Populations living in conflict-affected areas (e.g., war zones); Intervention/Exposure (I): Exposure to armed conflict; Comparison (C): Comparison between pre/post conflict period or conflict and non-conflict treated populations; Outcome (O): Vaccination coverage, immunization rates, access to vaccination services.*
