# Supplementary material for: The impact of armed conflict on vaccination coverage: a systematic review of empirical evidence from 1985 to 2025
Source: Confl Health. 2025 Oct 14;19:71. doi: 10.1186/s13031-025-00708-7 (PMC12519846; doi:10.1186/s13031-025-00708-7)
Supplement: Supplementary file 2 — Supplementary Material 2. [file 13031_2025_708_MOESM2_ESM.docx]

Additional Table 2: Detailed Characteristics and Results of the Included Studies

| **Citation** | **Year** | **Study Objective(s)** | **Country** | **Language** | **Conflict Data** | **Study Design** | **Vaccines Assessed** | **Immunization Data** | **Unit of Analysis** | **Main Effect Size** |
| --- | --- | --- | --- | --- | --- | --- | --- | --- | --- | --- |
| Akseer et al (1) | 2019 | To evaluate associations of conflict severity with improvement of health system performance, use of health services, and child nutrition outcomes in Afghanistan during the 2003 to 2018 reconstruction period. | Afghanistan | English | UCDP | Difference-in-differences | BCG, DPT3, Measles | Multiple Indicator Cluster Surveys and the 2018 Afghanistan Health Survey. | District | Moderate-to-severe conflict associated with lower vaccine coverage: BCG −3.8 pp/year (p=0.002), measles −2.9 pp/year (p=0.01), DPT3 −3.0 pp/year (p<0.001) compared to minimal conflict. |
| Al-Samhari et al (2) | 2023 | We conducted a nationwide retrospective observational study in Yemeni sentinel hospitals to identify the prevalence, aetiology, vaccination coverage and spatio-temporal pattern of ABM in children aged <5 years before and during the civil war, 2014–20. | Yemen | English | NA | nationwide retrospective observational study | HiB, PCV13 | EPI | Individual | Annual vaccination coverage declined: APC −1.92 by 2018. |
| Amberg et al (3) | 2023 | We aimed to establish how disruptions due to armed conflict affect health service coverage. | Sub-Saharan Africa | English | UCDP | Quasi-experimental using repeated cross-sectional DHS with fixed effects | BCG, DPT1, DPT2, DPT3, polio1, polio2, polio3, measles | DHS | Individual | Exposure to nearby armed conflict was associated with a -2.5 percentage point reduction in timely receipt of all basic vaccinations (95% CI -3.1 to -1.9). Longer conflicts (≥5 years) and higher intensity (≥1000 deaths) showed larger reductions of -5.9pp (95% CI -7.2 to -4.6) and -9.9pp (95% CI -13.3 to -6.6), respectively. |
| Batarfi et al (4) | 2024 | To investigate the effect of public health emergency crises on childhood immunization in Yemen, specifically in the Coastal Hadhramaut Governorate. | Yemen | English | Not explicitly stated; inferred through contextual references to the humanitarian crisis and internal displacement | Retrospective descriptive study | BCG, MR (measles-rubella), IPV, OPV, rotavirus, pentavalent (diphtheria, tetanus, pertussis, hepatitis B, Hib), PCV | Annual reports from the Health and Population Affairs Office – Coastal Hadhramaut; health facility records | District | Between 2015 and 2020, vaccination coverage in Coastal Hadhramaut declined across multiple vaccines. BCG fell by up to 15%, IPV by 14%, and MR coverage dropped intermittently, with only 4 of 12 districts achieving ≥80% MR1 coverage in 2020. Rotavirus and pentavalent vaccines declined by up to 9%, with similar decreases in OPV and PCV, reflecting broad disruptions from conflict and COVID-19. |
| Broholm et al (5) | 1989 | We report on a pilot study to assess the effects of low intensity war in Nicaragua on the health of the civilian population. | Nicaragua | English | Ministry of Health | cross-sectional observational | DPT, Polio, Measles, BCG | Primary survey | Household/child-level data aggregated by town | Children in the conflict-affected town of Acoyapa were significantly less likely to be fully vaccinated than those in the non-conflict town of El Ostional (OR 0.17, 95% CI 0.07–0.39). Immunisation card possession was also markedly lower in Acoyapa (OR 0.23, 95% CI 0.09–0.57). |
| Cetorelli, Valeria (6) | 2015 | This is the first study to examine whether, and how much, the Iraq war (2003–2011) impacted neonatal polio vaccination coverage. | Iraq | English | Implied from geographic and temporal war exposure | difference-in-differences | OPV | Iraq Multiple Indicator Cluster Surveys (I-MICS) from 2000, 2006, 2011 | Individual (child) | War-exposed children 21.5 pp less likely to receive neonatal polio vaccine (95% CI −34.1 to −8.9). |
| Chepkurui et al (7) | 2021 | This study assessed public health emergencies (armed conflicts, disasters, and disease outbreaks) and the performance of national immunization programs using global and regional immunization targets outlined for the Decade of Vaccines. | WHO Africa Region (13 beneficiary countries of PHE mitigation funds from the African Public Health Emergency Fund) | English | UCDP | case study | DPT3, DPT1 | WHO/UNICEF | National | Greater frequency of armed conflict and emergencies significantly associated with failure to reach 90% DPT3 coverage. |
| El Bcheraoui et al (8) | 2018 | This study aims to assess the impact of the ongoing war in Yemen on maternal and child health by providing a governorate-level situational analysis to inform response planning and resource allocation. | Yemen | English | number of airstrikes per 1000 population (shock variable) | retrospective, model-based ecological study | polio 3, DTP3, pneumococcal 3, measles 1 | DHS | governate | Coverage declined by 16.2% for DTP3, 17.9% for measles vaccine, 13.1% for pneumococcal vaccine, and 14.3% for polio3 (percent change, not percentage points). |
| Goli et al (9) | 2022 | We empirically estimate the relationship between exposure to conflict from in utero to age 5, and its association with child health outcomes using a sam- ple of 590,488 pre-school age children across 52 coun- tries over the period 1997 to 2018. | 52 developing countries | English | UCDP | Multi-country pooled repeated cross-sectional study with quasi-experimental approach | BCG, Measles, DPT1, DPT2, DPT3, polio1, polio2, polio3 | DHS | Individual | Exposure to conflict associated with reductions in full vaccination: −0.4% per conflict year; −2.3 to −2.8% for high mortality conflicts. |
| Grossman et al (10) | 2019 | To study how the recent rise in terrorist activity affects health of children exposed to violence. | Pakistan | English | Global Terrorism Database | quasi-experimental | tuberculosis and pentavalent | MICS (2011) | individual/household | A one SD increase in attack intensity before birth was associated with 2–7 fewer children per 1,000 receiving the tuberculosis vaccine. Exposure during the final two to three months of pregnancy resulted in 7–8 fewer children per 1,000 being vaccinated. |
| Haddison et al (11) | 2020 | We assessed the utilisation of health services before and during the armed conflicts in the Southwest region of Cameroon. | Cameroon | English | NA | Retrospective observational study using routine health data. | BCG, DTP3 | EPI registers | regional | DPT3 dropped from 90% (2017) to 55% (2018); BCG from 87% (2017) to 54% (2018). |
| Jawad et al (12) | 2021 | This study assesses the association between conflict and maternal and child health globally. | Worldwide | English | UCDP | panel (longitudinal) study | DPT, measles | World Bank | National | Armed conflict was associated with a 2.6% (95% CI 0.2–5.0) reduction in measles vaccine coverage, while wars were linked to 4.9% (95% CI 1.5–8.3) and 7.3% (95% CI 2.7–11.8) reductions in DPT and measles coverage, respectively. These effects persisted for up to three years (DPT) and two years (measles) following war onset. |
| Kreif et al (13) | 2022 | This paper investigates the causal impacts of the long-term internal conflict on child health in Colombia, following an identification strategy based on the temporal and geographic variation of conflict intensity. | Colombia | English | Centro Nacional de Momoria Historica data (Colombian conflict data), Centro de Recursos para el Analisis de Conflictos (presence of armed groups) | cross-sectional, pseudo-panel cohort | "full childhood vaccination" | DHS | Household | No statistically significant association between conflict and vaccination coverage. |
| Leone et al (14) | 2019 | This study assesses the relationship between conflict intensity and access to Maternal and Child Health care in occupied Palestinian territory. | Occupied Palestinian Territory (oPt) | English | B’Tselem (2016) | pseudo-longitudinal analysis | DPT, OPV | DHS (2004), Palestinian Family Health Survey (2006), Palestinian Family Survey (2010), Palestinian Multiple Indicator Cluster Survey (2014) | Individual | Conflict intensity negatively associated with OPV: β=−0.223 (p<0.001); and DPT: β=−0.227 (p<0.001). |
| Malembaka et al (15) | 2021 | We aimed to describe the levels and trends in RMNCH indicators within Kivu provinces between 2015 and 2018, by linking conflict data with health facility (HF) data from the District Health Information System 2 (DHIS2). | Kivu, DR Congo | English | UCDP | Retrospective time-series with fixed-effects regression | DPT1, DPT3 | DHIS2 (routine health facility data); 2014 DHS; 2018 MICS | Health zone level | Under the most inclusive conflict threshold, the estimated effect on DPT3 coverage was 1.35 (95% CI −1.67 to 4.37), indicating a non-significant positive association between severe conflict and DPT3 coverage. |
| Mashal et al (16) | 2007 | To evaluate progress and variation in infant immunization coverage by district and region in Afghanistan and assess the impact of conflict and resource availability on immunization coverage. | Afghanistan | English | Expert focus group discussions; subjective regional ratings (not from ACLED or UCDP). | Cross-sectional (with pre-post comparison at two time points: 2000 and 2003); multivariate logistic regression applied. | BCG, DPT3, OPV3, Measles | Reports from EPI centers (district-level data from Ministry of Public Health, WHO, and UNICEF) | District | Compared with highly insecure regions, occasionally insecure areas had significantly higher odds of achieving ≥80% vaccination coverage for BCG (OR 3.13, 95% CI 1.56–6.31, p<0.001), DPT3 (OR 12.16, 95% CI 3.40–43.55, p<0.001), OPV3 (OR 2.99, 95% CI 1.21–7.42, p=0.02), and measles (OR 10.31, 95% CI 2.71–39.23, p<0.001). In insecure regions, odds were higher for DPT3 (OR 4.17, 95% CI 1.06–16.38, p=0.04) and OPV3 (OR 3.09, 95% CI 1.03–9.29, p=0.04), but not significant for BCG or measles. |
| Masset, Edoardo (17) | 2022 | We estimate the impact of the Malian conflict on child mortality over the period 2012–2018 using Demographic Health Survey data. | Mali | English | Implied from regional classification; not explicitly reported | Synthetic Difference-in-Differences (SDID) | two tetanus vaccinations; full vaccination cycle | DHS | Region | In a comparison between conflict-affected North and non-conflict South, vaccination coverage was 8% lower in the North (SE = 0.041). |
| Mezen et al (18) | 2023 | To assess the association of war with vaccination dropout among children younger than 2 years in the North Wollo zone of Ethiopia. | Ethiopia | English | Primary Survey | cross-sectional | Composite (full vaccination of BCG, pentavalent, polio, measles) | Primary Survey | Household | 44% of children initiating vaccination before war dropped out; conflict duration inversely associated with dropout: OR 1.75 (95% CI 1.11–2.77) for 1-month exposure; OR 0.51 (95% CI 0.28–0.93) for 5-month exposure. |
| Mirzazada et al (19) | 2020 | We aimed to examine the direct impact of conflict on the delivery of reproductive, maternal, newborn, child, and adolescent health and nutrition (RMNCAH&N) services, and to identify the contextual factors that influence these services. | Afghanistan | English | UCDP, Delphi methodology with expert input | Cross-sectional (quantitative) + qualitative interviews | DPT3, BCG, Measles | DHS, MICS | Province (for conflict classification); individuals (for outcomes) | DPT3 coverage was associated with a 74% decrease (OR 0.26, 95% CI 0.20–0.33), BCG coverage with a 180% increase (OR 2.80, 95% CI 2.09–3.76), and measles coverage showed a non-significant 36% increase (OR 1.36, 95% CI 0.95–1.94). |
| Mohamed et al (20) | 2022 | We aimed to explore the level of antenatal tetanus vaccination and to identify the influencing factors in a nationally representative population sample. | Sudan | English | Scale created by Heidelberg Institute for International Conflict Research, which measures intensity based on weapons, personnel, casualties, refugees & IDPs, and destruction. Barometer, Conflict. "disputes non-violent crises violent crises limited wars wars, 26." Heidelberg Institute for International Conflict Research, Heidelberg (2018): 108. | cross-sectional | tetanus | Sudan Multiple Indicator Cluster Survey | Individual | Low conflict intensity associated with higher odds of antenatal tetanus vaccination than other areas: AOR 1.34 (95% CI 1.14–1.57). |
| Naufal et al (21) | 2020 | To assess the impact of conflict on childhood vaccination rates in Iraq using a generalized difference-in-differences approach. | Iraq | English | Iraq Body Count (IBC) project (media sources, NGO, and government records). | Generalized difference-in-differences (quasi-experimental). | BCG, Hepatitis B, DTP/Pentavalent, Measles/MMR, Polio | Multiple Indicator Cluster Survey (MICS) from 2006, 2011, and 2018. | Individual child | Probit coefficients for high-conflict exposure were 0.178 for any vaccine (p < 0.01), 0.123 for BCG (p < 0.05), and 0.090 for measles/MMR (p < 0.05), indicating significant positive associations; no significant effects were observed for polio, Hep B, DPT, or pentavalent vaccines. |
| Ojeleke et al (22) | 2022 | The aim of this study is to explore the effect of protracted armed conflicts on healthcare utilization, measured by maternal care and child immunization, in Northern Nigeria. | Nigeria | English | ACLED | Difference-in-differences | Not specified (child has ever been immunized) — no vaccine types mentioned. | DHS | individual-level nested within clusters | Child immunization odds were reduced by 14–19% within 5 km (OR 0.81–0.86) and 18–38% within 10 km (OR 0.62–0.82) of conflict, paralleling declines seen in antenatal care and facility births. |
| Omer et al (23) | 2014 | We explored the effects on health of both household asset inequality and political armed conflict in Sudan. | Sudan | English | Not specified; conflict status defined by authors based on geographic knowledge (Darfur and Blue Nile) | cross-sectional | full vaccination (polio, tuberculosis, diphtheria, tetanus, pertussis, hepatitis B, mumps and measles) | Sudan Household Health Survey-2 | State | Children in conflict-affected states had 4.5 percentage points lower full vaccination rates. No confidence intervals or p-values were reported. |
| Østby et al (24) | 2021 | This article studies how armed conflict impacts immunization among children. | Sub-Saharan Africa (15 countries) | English | UCDP | cohort, quasi-expiremental | BCG, Polio 1, Polio 2, Polio 3, DPT1, DPT2, DPT3 | DHS | Household | Minor conflict associated with slight increase in immunization (+0.002); major conflict associated with modest decrease (−0.001); 100 BRD increase associated with −0.004 probability of full immunization. |
| Ruiz, Gladys Cecilia Ghisays (25) | 2003 | The aim is to quantify the degree of correlation between poverty, rural population density, health reform, the presence of armed conflict, and the level of decentralization in municipalities, and their corresponding impact on the reduction in vaccine coverage. | Colombia | Spanish | government records | ecological study | BCG, DPT (DPT3 is tracer), OPV, SPR (measles) | EPI | municipality | Municipalities where the armed conflict was present had 7% less coverage than municipalities where the conflict was not present. |
| Saidu et al (26) | 2021 | We examined how the conflict affected important vaccination outcomes in the North West and South West regions of Cameroon. | Cameroon | English | NA | Repeated cross-sectional/pre-post | BCG, DPT-3, MR, PCV-13, Rotarix-2, OPV | EPI District Vaccine and Data Management Tool | health district | BCG and DPT3 coverage declined by 22 pp and 42 pp, respectively (2016–19); similar trends for PCV13, IPV, and Rotavirus vaccines. |
| Sato, Ryoko (27) | 2019 | To compare the relative effect of the Boko Haram insurgency, as compared to other conflict events, on childhood vaccination uptake in Nigeria. | Nigeria | English | UCDP | cross-sectional | BCG, Pentavalent 3 (DPT3) | DHS | Household | Conflict events during or shortly after childbirth reduced odds of any vaccination by 41.9%–48.0%, and conflict events within two months of childbirth were associated with up to 59% lower likelihood for BCG and 56.1% for DPT1. No significant effect was observed when conflict occurred three months after birth. |
| Sato, Ryoko (28) | 2021 | We evaluate the impact of armed conflict on children's vaccination rates. | Nigeria | English | UCDP | Cross-sectional with quasi-experimental elements (difference-in-differences, logistic regression with temporal variation in exposure). | BCG, DTP1 | DHS | Individual child (clustered by geographic location) | In Boko Haram–affected areas (Yobe, Adamawa, and Borno), conflict exposure was associated with a 35% reduction in the odds of a child ever being vaccinated (OR 0.65, 95% CI 0.44–0.97, p<0.01), with stronger negative effects observed for DPT3/Penta3 uptake (OR 0.61, 95% CI 0.41–0.91, p<0.01). The effect on BCG vaccination was smaller and not statistically significant (OR 0.97, 95% CI 0.66–1.43). Across Nigeria overall, conflict exposure was linked to a 26% increase in odds of any vaccination (OR 1.26, 95% CI 1.01–1.56, p<0.05) and a 52% increase in BCG vaccination (OR 1.52, 95% CI 1.24–1.86, p<0.001), with no significant effect on DPT3/Penta3 uptake (OR 1.05, 95% CI 0.90–1.23). |
| Schaub et al (29) | 2025 | To assess the impact of individual-level violent victimization on maternal and child healthcare-seeking behavior in northeastern Nigeria, and to identify mechanisms (e.g., mistrust, fear, PTSD) linking victimization to outcomes. | Nigeria | English | Primary survey data based on self-reported victimization from armed conflict | Matched case-control design with causal mediation analysis. | DPT3, Measles (as referenced in matching variables); general full childhood immunization schedule as per Nigerian Ministry of Health | Primary household survey, verification using immunization card | Individual (caregiver–child dyads) | The odds of full immunization were reduced by 57% (OR 0.43, 95% CI 0.32–0.57). |
| Tewantsa et al (30) | 2024 | To examine the impact of the socio-political crisis on the performance of the Expanded Program on Immunization (EPI) in the North West and South West regions of Cameroon. | Cameroon | English | Not explicitly reported; inferred from regional health system and national reports. | Secondary data analysis / natural experiment (pre/post comparison). | BCG, PENTA (PENTA1 and PENTA3), RR (measles-rubella) | EPI | District | In the North West, PENTA3 coverage decreased by 7% (Z=3.40, p<0.001) and RR1 by 8% (Z=4.14, p<0.001). In the South West, coverage declined by 22% for BCG, 33% for PENTA3, and 36% for RR1 (all p<0.001). |
| Torbosh et al (31) | 2019 | The aim of this study was to determine the impact of the 2015 war on the immunization coverage of children under 1 year. | Yemen | English | NA | cross-sectional | penta-3, measles, BCG | Expanded Program on Immunization | governate/national | National measles coverage declined from 75% to 66% (2014–15); Penta-3 from 88% to 84%; BCG from 73% to 49%, especially in conflict-affected governorates. |
| Verma et al (32) | 2018 | To examine the hypothesis that insecurity is associated with reduced polio vaccination and increased paralytic polio incidence in northwest Pakistan, and that this relationship is mediated by vaccinator access. | Pakistan | English | Journalistic reports aggregated by Pakistan Body Count (PBC), validated with PIPS and Bureau of Investigative Journalism (BIJ) | Natural experiment; longitudinal observational study using multilevel Poisson regression with fixed effects. | Polio (Oral Polio Vaccine) | Routinely collected health data from 666 district-level vaccination campaigns | District-level vaccination campaign; district-month for polio incidence | Vaccination coverage was 5.3% lower (95% CI 5.2–5.3) in high-insecurity campaigns compared with secure campaigns. |
| Zhang et al (33) | 2023 | Using a theoretical utility model and an empirical analysis of panel data from multiple countries, this study aims to answer the core question: What effects does armed conflict have on maternal health-seeking behavior (MHSB)? | Chad, CAR, DRC, and Iraq | English | UCDP | Difference-in-differences with PSM (propensity score matching) | tetanus vaccination | UNICEF Multiple Indicator Cluster Surveys (MICS4 and MICS6) | Individual | Armed conflict positively associated with maternal tetanus vaccination: β=0.055 (95% CI 0.004–0.106; p<0.05). |

**FULL CITATIONS**

1. Akseer N, Rizvi A, Bhatti Z, Das JK, Everett K, Arur A, et al. Association of exposure to civil conflict with maternal resilience and maternal and child health and health system performance in Afghanistan. JAMA Network Open. 2019;2(11):e1914819-e.

2. Al-Samhari GA, Al-Mushiki GM, Tamrakar R, Lin Y-D, Al-Shaebi F, Akroot MA, et al. Prevalence, aetiology, vaccination coverage and spatio-temporal pattern among patients admitted with acute bacterial meningitis to the sentinel hospital surveillance network in Yemen, 2014–20, before and during the civil war. International journal of epidemiology. 2023;52(4):1175-86.

3. Amberg F, Chansa C, Niangaly H, Sankoh O, De Allegri M. Examining the relationship between armed conflict and coverage of maternal and child health services in 35 countries in sub-Saharan Africa: a geospatial analysis. The Lancet Global Health. 2023;11(6):e843-e53.

4. Batarfi SA, Sutan R, Ismail H, Bin-Ghouth AS. Immunization of Children under 2 Years Old in the Coastal Hadhramaut Governorate, Yemen, during Public Health Emergencies: A Trend Analysis of 2013–2020. Vaccines. 2024;12(3):311.

5. Broholm C, Clements C, DiPrete L, Epstein P, Fine A, Halloran M. Health effects of the war in two rural communities in Nicaragua. 1989.

6. Cetorelli V. The impact of the Iraq War on neonatal polio immunisation coverage: a quasi-experimental study. J Epidemiol Community Health. 2015;69(3):226-31.

7. Chepkurui V, Amponsah-Dacosta E, Haddison EC, Kagina BM. Characterization of national immunization programs in the context of public health emergencies: A case study of 13 countries in the WHO Africa region. Frontiers in public health. 2021;9:736532.

8. El Bcheraoui C, Jumaan AO, Collison ML, Daoud F, Mokdad AH. Health in Yemen: losing ground in war time. Globalization and health. 2018;14:1-12.

9. Goli S, Mavisakalyan A, Rammohan A, Vu L. Exposure to conflict and child health outcomes: evidence from a large multi-country study. Conflict and health. 2022;16(1):52.

10. Grossman D, Khalil U, Ray A. Terrorism and early childhood health outcomes: evidence from Pakistan. Social Science & Medicine. 2019;237:112453.

11. Haddison EC, Julius CE, Kagina BM. Health services utilisation before and during an armed conflict; experiences from the southwest region of Cameroon. The Open Public Health Journal. 2020;13(1).

12. Jawad M, Hone T, Vamos EP, Cetorelli V, Millett C. Implications of armed conflict for maternal and child health: a regression analysis of data from 181 countries for 2000–2019. PLoS Medicine. 2021;18(9):e1003810.

13. Kreif N, Mirelman A, Suhrcke M, Buitrago G, Moreno-Serra R. The impact of civil conflict on child health: Evidence from Colombia. Economics & Human Biology. 2022;44:101074.

14. Leone T, Alburez-Gutierrez D, Ghandour R, Coast E, Giacaman R. Maternal and child access to care and intensity of conflict in the occupied Palestinian territory: a pseudo-longitudinal analysis (2000–2014). Conflict and health. 2019;13:1-15.

15. Malembaka EB, Altare C, Bigirinama RN, Bisimwa G, Banywesize R, Tabbal N, et al. The use of health facility data to assess the effects of armed conflicts on maternal and child health: experience from the Kivu, DR Congo. BMC health services research. 2021;21:1-11.

16. Mashal T, Nakamura K, Kizuki M, Seino K, Takano T. Impact of conflict on infant immunisation coverage in Afghanistan: a countrywide study 2000–2003. International Journal of Health Geographics. 2007;6:1-9.

17. Masset E. Conflict and child mortality in Mali: A synthetic control analysis. Population and Development Review. 2022;48(4):1097-123.

18. Mezen MK, Lemlem GA, Biru YB, Yimer AM. Association of War with Vaccination Dropout among children younger than 2 years in the North Wollo Zone, Ethiopia. JAMA network open. 2023;6(2):e2255098-e.

19. Mirzazada S, Padhani ZA, Jabeen S, Fatima M, Rizvi A, Ansari U, et al. Impact of conflict on maternal and child health service delivery: a country case study of Afghanistan. Conflict and health. 2020;14:1-13.

20. Mohamed SOO, Ahmed EM. Prevalence and determinants of antenatal tetanus vaccination in Sudan: a cross-sectional analysis of the Multiple Indicator Cluster Survey. Tropical Medicine and Health. 2022;50:1-6.

21. Naufal G, Malcolm M, Diwakar V, editors. Violent conflict and vaccinations: Evidence from Iraq2020: Economic Research Forum (ERF).

22. Ojeleke O, Groot W, Bonuedi I, Pavlova M. The impact of armed conflicts on health‐care utilization in Northern Nigeria: A difference‐in‐differences analysis. World Medical & Health Policy. 2022;14(4):624-64.

23. Omer AS, Bezruchka S, Longhi D, Kelly Z, Brown M, Hagopian A. The effects of household assets inequality and conflict on population health in Sudan. African Population Studies. 2014;28(3):1216-32.

24. Østby G, Shemyakina O, Tollefsen AF, Urdal H, Verpoorten M. Public health and armed conflict: immunization in times of systemic disruptions. Population and Development Review. 2021;47(4):1143-77.

25. Ruiz GCG. Variables relacionadas con las coberturas de vacunación en los municipios colombianos, 2000. Avances en Enfermería. 2003;21(2):44-53.

26. Saidu Y, Vouking M, Njoh AA, Bachire HB, Tonga C, Mofor R, et al. The effect of the ongoing civil strife on key immunisation outcomes in the North West and South West regions of Cameroon. Conflict and health. 2021;15:1-8.

27. Sato R. Effect of armed conflict on vaccination: evidence from the Boko haram insurgency in northeastern Nigeria. Conflict and health. 2019;13:1-10.

28. Sato R. Differential effect of conflicts on vaccination: Boko Haram insurgency vs. other conflicts in Nigeria. Medicine, Conflict and Survival. 2021;37(4):275-92.

29. Schaub M, Adeyanju GC, Abulfathi AA, Bello MM, Kasserra L, Kwaku AA, et al. Maternal and child healthcare-seeking among victims of violence in armed conflict: A matched case-control study in northeast Nigeria. medRxiv. 2025:2025.03. 11.25323760.

30. Tewantsa JM, Bekolo CE, Enyama D, Mabou GT, Choukem SP. Impact of the Socio-political Crisis in the North West and South West Regions of Cameroon on the Expanded Vaccination Program for Children From 0 to 23 Months. 2024.

31. Torbosh A, Al Amad MA, Al Serouri A, Khader Y. The impact of war in Yemen on immunization coverage of children under one year of age: descriptive study. JMIR Public Health and Surveillance. 2019;5(4):e14461.

32. Verma AA, Jimenez MP, Tangermann RH, Subramanian S, Razak F. Insecurity, polio vaccination rates, and polio incidence in northwest Pakistan. Proceedings of the National Academy of Sciences. 2018;115(7):1593-8.

33. Zhang T, He Q, Richardson S, Tang K. Does armed conflict lead to lower prevalence of maternal health-seeking behaviours: theoretical and empirical research based on 55 683 women in armed conflict settings. BMJ Global Health. 2023;8(8):e012023.
